# Patient Characteristics and Preferences Regarding Anticoagulant Treatment in Venous Thromboembolic Disease

**DOI:** 10.3389/fcvm.2021.675969

**Published:** 2021-06-21

**Authors:** Damien Lanéelle, Charles Le Brun, Chadi Mauger, Jérôme Guillaumat, Estelle Le Pabic, Loukman Omarjee, Guillaume Mahé, SFMV VTE Study Group

**Affiliations:** ^1^Centre Hospitalier Universitaire Caen Normandie, Service de Médecine Vasculaire, Caen, France; ^2^Université de Caen, COMETE, Caen, France; ^3^Centre Hospitalier Universitaire de Rennes, Department of Vascular Medicine, Rennes, France; ^4^Université de Rennes 1, Rennes, France

**Keywords:** patient preference (MeSH), venous thromboembolism disease (VTE disease), patient concern, anticoagulation/administration and dosing, anticoagulation management

## Abstract

**Background:** Anticoagulants are the recommended treatment for venous thromboembolic disease (VTE). The mode of anticoagulant administration may influence compliance, and therefore the effectiveness of the treatment. Unlike in atrial fibrillation or cancer-associated thrombosis, there is only limited data on patient preferences regarding the choice of anticoagulation in VTE. This study aims to evaluate patient preferences regarding anticoagulants in terms of administration: types (oral or injectable treatment) and number of doses or injections per day.

**Patients and Methods:** This is a national survey through a questionnaire sent by e-mail to 1936 French vascular physicians between February and April 2019. They recorded the responses for each patient admitted for VTE.

**Results:** Three hundred and eleven (response rate of 16%) of the 1936 contacted physicians responded for 364 patients. Among these, there were 167 fully completed questionnaires. Most patients (63%) express concerns about VTE and prefer oral treatment (81.5%), justified by the ease of administration (74%) and a fear of the injections (22%). When patients were taking more than three oral treatments they statistically chose injectable treatment more often (54%) than oral treatment (25%, *p* = 0.002). Patients who chose injectable treatment were also older (70 ± 16 vs. 58 ± 17 years old, *p* = 0.001). There was no statistically difference in anticoagulation preference according to gender or to the expected duration of treatment (6 weeks, 3 months, 6 months or unlimited). When oral treatment was preferred (81%), most chose oral treatment without dose adjustment and biomonitoring (74.3%). Among them, very few (5.8%) preferred a twice-daily intake.

**Conclusion:** Patient preference in terms of anticoagulant treatment in VTE disease is in favor of oral treatment without adjustment or biomonitoring and with once-daily intake. When an injectable treatment is chosen, a prolonged duration of treatment does not seem to be a constraint for the patient.

**Clinical Trial Registration:** ClinicalTrials.gov, identifier [NCT03889457].

## Keypoint

- Patients with venous thromboembolic disease prefer oral treatment without adjustment or biomonitoring and with once-daily intake.
- Patients who chose injectable treatment were older and taking more than three oral treatments.
- When injectable anticoagulation treatment is indicated (e.g., cancer-associated thrombosis), prolonged duration does not seem to be a constraint for the patient.

## Introduction

Venous thromboembolic disease (VTE), including pulmonary embolism (PE), deep venous thrombosis (DVT) and superficial venous thrombosis (SVT), is a major burden with an incidence of ~10 million cases yearly, thereby representing the third leading vascular disease after myocardial disease and stroke (1). In case of provoked proximal DVT or PE, 3 months of oral therapeutic anticoagulation is currently recommended, usually by oral treatment (vitamin K antagonist VKA, or direct oral anticoagulants DOAC) or by injectable treatment (2).

A previous study suggests that long-term injectable anticoagulation is an acceptable alternative to oral anticoagulation for the patient, whereas physicians underestimate the acceptability of this mode of administration (3). The mode of administration may influence compliance, and therefore the effectiveness of treatment but, unlike atrial fibrillation (4) or cancer-associated thrombosis (5), there is only limited data on patient preferences regarding the choice of anticoagulation treatment.

The main objective of this study is to explore patient preferences in terms of administration modality: types (oral or injectable treatment) and number of intakes or injections per day. The secondary objective is to analyze the external factors (medical history, age, gender, social environment, personal experience or expected treatment duration) that may influence this choice.

## Materials and Methods

This national survey was drawn up by a questionnaire with three parts: patient demographics (age, weight, gender, concern about VTE, familial or personal VTE history, number of treatment); clinical situations (DVT, PE or SVT); inpatient or outpatient; associated risk factors and preferences in terms of administration modality: oral or injectable treatment and frequency of intake (once/twice daily). We also studied the justification (ease of administration, apprehension, or other) and the choice of patient according to the expected duration of treatment (6 weeks, 3 months, 6 months or unlimited) as well as the preference for biological monitoring with dose adjustment rather than a fixed dose. The French Society of Vascular Medicine (SFMV) emailed the questionnaire to members who recorded the responses for each patient admitted for VTE. It was a face-to-face interview with a standardized questionnaire. Physicians were instructed not to add any additional information to the questionnaire text in order to avoid introducing bias. The physician presented the information and presentation of the medical options orally in a standardized manner using a typical sentence like “There are two types of treatment: oral or injectable treatment with needle made by yourself or a nurse. Both are “good” (i.e., recommended) treatments, what type of treatment would you prefer if you could choose?” (Appendix 1). Vascular physicians collected the data either by filling out a paper questionnaire or an online questionnaire using Limesurvey software (LimeSurvey GmbH, Hambourg) only in patients who signed the informed consent to participate. The estimated time for answering questions was 5 min. Anonymous, data were centralized in a database hosted at the Rennes University Hospital. Participation was not paid. The full questionnaire is presented in the Appendix 1.

The study was declared on ClinicalTrials.gov (NCT03889457) and the Ethics Committee of the University of Rennes (CPP #8/589) approved the protocol. Using information recorded on the case report forms, we classified the index VTE as provoked by (a) persistent risk factors (active cancer, inflammatory bowel disease, varicose veins, or known thrombophilia); (b) transient risk factors (surgery or trauma, catheter, immobilization, travel >8 h, pregnancy, puerperium, or use of estrogen). The details of this classification are included in the Appendix 2. Patients without any of these risk factors were classified as having unprovoked VTE (6).

### Statistical Analysis

Quantitative results are expressed in mean ± standard deviation and qualitative results (concerns about VTE, familial or personal VTE history, number of treatment, type of VTE, inpatient, and associated risk factors) are expressed in percentage (%). For group comparisons, according to preferences in terms of administration modality, the Student's parametric test or Mann-Whitney Wilcoxon's non-parametric test was used for quantitative variables and the Chi2 test or Fisher's exact test was used for qualitative variables. All statistical tests have a significance level of 0.05. Statistical analyses were performed using SAS software, v.9.4® (SAS Institute, Cary, NC, USA).

## Results

Between February 2019 and November 2019, 1936 e-mails were sent to all French Society of Vascular Medicine (SFMV) members. Three hundred and eleven (response rate of 16%) of the 1936 contacted physicians responded for 364 patients. Among them, 197 were incomplete questionnaires and were excluded. Thus, there were 167 fully completed questionnaires.

Characteristics of the 167 patients are shown in Table 1. The average age was 61 years ±14 with 59% male. The thrombotic event was mostly DVT (80%), then PE (33%) and SVT (10%); 4% had SVT associated with DVT and 18% DVT associated with PE. Among DVT, 97% was on lower limbs. In 32% of patients, the thrombotic event was unprovoked; in 23%, it was associated with a permanent risk factor and in 59% with a transient risk factor. Most patients (51%) express concerns about VTE.

**Table 1:** Characteristics of the 145 patients.

**Variable**	**Value (percentage)**
Sex (male)	98 (59%)
Age (years ± SD)	61 ± 14
Inpatient	88 (53%)
Personal history of VTE	47 (28%)
History of injectable anticoagulation	9 (5%)
History of oral anticoagulation	12 (7%)
History of both type of anticoagulation	24 (14%)
No anticoagulation history	2 (1%)
History of VTE in a family member or friend	66 (40)%
**Number of prescription**	
0	38 (23%)
1–3	78 (47%)
> 3	51 (30)
Feeling overmedicated	44 (26%)
Concerned about the diagnosis	86 (51%)
**Thrombotic event**	
DVT	133 (80%)
DVT of lower limb	129 (77%)
PE	55 (33%)
SVT	16 (10%)
DVT + SVT	7 (4%)
DVT + PE	30 (18%)
VTE unprovoked	54 (32%)
VTE provoked by Transient risk factor (<3 months)	98 (59%)
Recent surgery with general anesthesia	21 (13%)
Lower limb trauma	8 (5%)
Immobilization (> 3 days)	44 (26%)
Oral contraceptive	6 (4%)
Long distance plane travel	21 (13%)
Catheter-related	5 (3%)
Pregnancy/post-partum	5 (3%)
Hormonal replacement therapy	4 (2%)
VTE provoked by Permanent risk factor	39 (23%)
Active Cancer	29 (17%)
Thrombophilia	7 (4%)
Chronic inflammatory disease	6 (4%)
Varicose veins	9 (5%)

*VTE, venous tromboembolism; PE, pulmonary embolism; DVT, deep venous thrombosis; SVT, superficial venous thrombosis; SD, standard deviation*.

Regarding the choice of treatment, a majority of patients prefer oral treatment (81.5%). Other patients prefer injectable treatment (8.4%), or have no preference (10.1%, Table 2).

**Table 2:** Patient preferences in terms of administration modality: dosage form and number of intakes per day.

**Dosage form**	**Value (percentage)**
Oral treatment with dose adjustment and biological monitoring	12 (7.2%)
Oral treatment without adjustment or biological monitoring	124 (74.3%)
Injectable treatment	14 (8.4%)
No preference	17 (10.1%)
**If oral treatment, justification**	
Ease of administration	101 (74.0%)
More efficient than injections	0 (0.0%)
Fear of injections/dont like injection	30 (22.0%)
Too many injection	0 (0.0%)
Less forgetting to take medication	0 (0.0%)
Other/not justified	5 (4.0%)
**If oral treatment, number of intakes per day**	
Twice a day	8 (5.8%)
Once a day	69 (50.0%)
No preference	61 (44.2%)
**If injectable treatment, justification**	
Ease of administration	0 (0.0%)
More efficient than pills	6 (42.8%)
Fear of pills/dont like pills	0 (0.0%)
Too many pills	6 (42.8%)
Less forgetting to take medication	2 (14.4%)
Other/not justified	0 (0.0%)
**If injectable treatment, number per day**	
Twice a day	1 (7.1%)
Once a day	9 (64.2%)
No preference	4 (28.7%)

The choice of oral administration was justified by the ease of administration (74%) and a fear of the injections (22%). The choice of injection was justified by a feeling of greater efficiency (42.8%), the wish not to take an additional oral treatment (42.8%) and the fear of forgetting (14.4%). When oral treatment was preferred (81.5%), most of patients chose oral treatment without dose adjustment and biomonitoring (74.3%) rather than with dose adjustment and biomonitoring (7.2%). Among them, very few (5.8%) preferred twice-daily intake.

**Figure 1:**
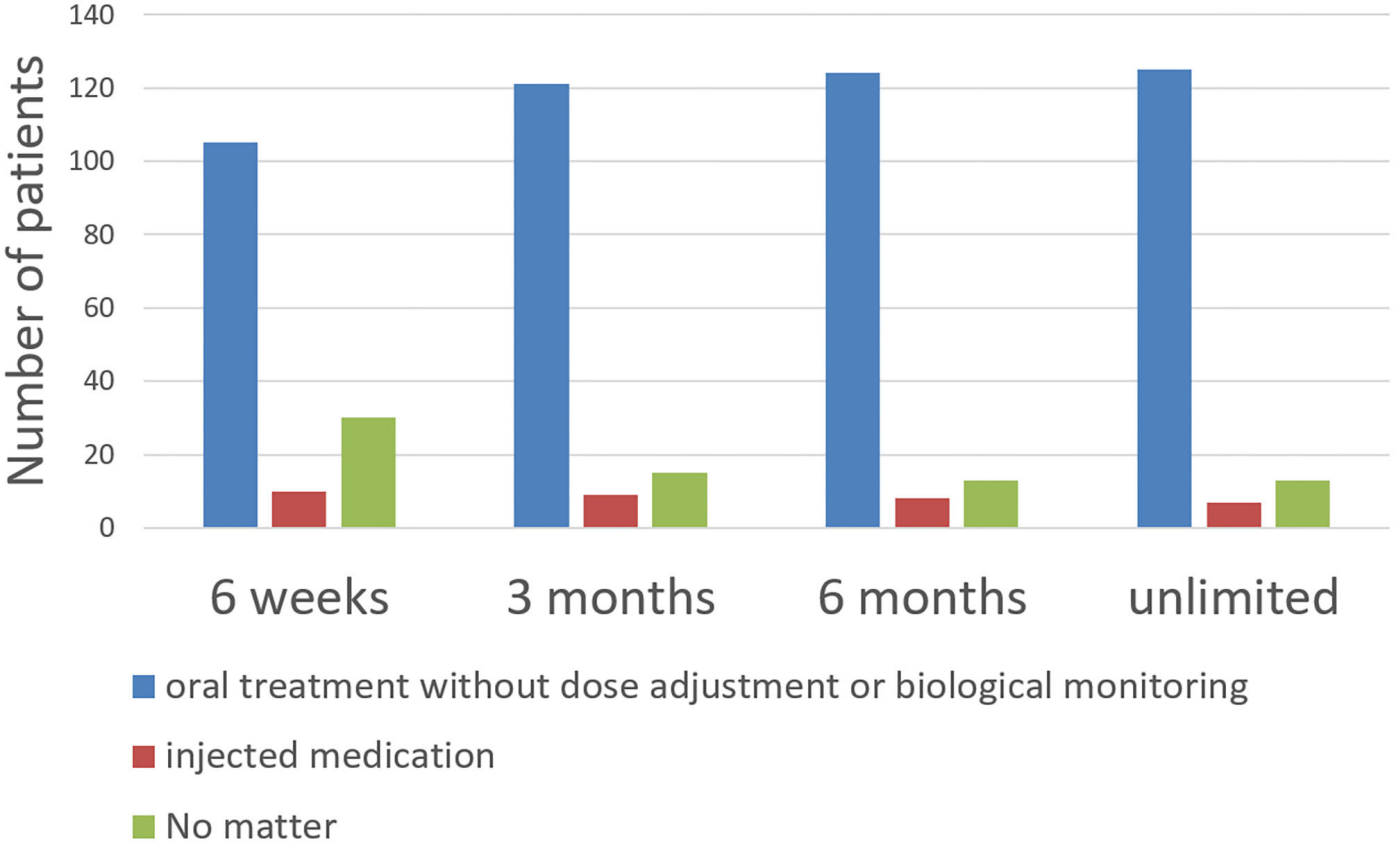
Patient preferences in terms of administration modality according to expected duration of treatment.

When patients were taking more than three oral treatments they statistically chose injectable treatment more often (54%) than oral treatment (25%, *p* = 0.002, Table 3) while when they took <3 oral treatment they more often chose oral treatment (52 vs. 23%). Patients who chose injectable treatment were also older (70 ± 16 vs. 58 ± 17 years old, *p* = 0.001) and less frequently had a PE (13 vs. 38%, *p* = 0.016). There was no statistically significant difference in anticoagulation preference according to age or gender or the expected duration of treatment (6 weeks, 3 months, 6 months or unlimited, Figure 1).

**Table 3:** Influence of patient characteristics and thrombotic event in the choice of oral or injectable anticoagulant therapy.

**Characteristics**	**Choice of anticoagulant therapy**		
	**Oral *n* = 136**	**Injectable or no preference *n* = 31**	***p*-value**
Sex (male)	79 (58%)	19 (61%)	0.901
Age (years ± SD)	58 ± 17^*^	70 ± 16^*^	0.001
Inpatient	69 (51%)	19 (61%)	0.388
History of injectable AC	8 (6%)	1 (3%)	0.880
History of oral AC	11 (8%)	1 (3%)	0.575
History of VTE in a relative	51 (38%)	15 (48%)	0.360
Number of prescriptions			
0	31 (23%)	7 (23%)	1.000
1–3	71 (52%)^*^	7 (23%)^*^	0.005
> 3	34 (25%)^*^	17 (54%)^*^	0.002
Feeling overmedicated	32 (24%)	12 (39%)	0.132
Concerned about the diagnoses	68 (50%)	18 (58%)	0.541
Thrombotic event			
DVT	104 (76%)	29 (93%)	0.060
PE	51 (38%)^*^	4 (13%)^*^	0.016
SVT	14 (10%)	2 (6%)	0.751
VTE unprovoked	43 (32%)	11 (35%)	0.839
VTE provoked by Transient risk factor (<3 months)	79 (58%)	19 (61%)	0.901
VTE provoked by Permanent risk factor	33 (24%)	6 (19%)	0.728

*AC, anticoagulation; VTE, venous tromboembolism; PE, pulmonary embolism; DVT, deep venous thrombosis; SVT, superficial venous thrombosis; SD, standard deviation*.

* *p < 0.05*.

There was no difference in anticoagulation preference between patients with DVT or SVT diagnosis and no difference between outpatients and inpatients. There was no statistically difference in preference according to history of VTE (personal or family), concern about VTE or to associated risk factors.

## Discussion

Patient satisfaction to treatment and patient preference result in better adherence to treatment and an overall reduction in the incidence of recurrent thrombotic event and bleeding risk (7). Recommendations emphasize that the choice of anticoagulant for treatment of VTE depends, especially for unlimited anticoagulation, on the patient's preferences, which is important for the acceptance of treatment and, therefore, for adherence and persistence (2). However, knowledge on this subject is sparse, particularly for patients with VTE in a non-cancer context and patient with unprovoked VTE for which half-dose of DOAC use is increasing (8). Indeed, in a cancer-associated thrombosis, some qualitative studies have focused on patients' preference regarding anticoagulant treatment (5, 9). In these patients, VKA is not recommended and injectable treatment was considered an acceptable intervention despite challenges of long-term injections with bruising and deterioration of injection sites. Participants would only favor a DOAC if it was equivalent to injected treatment (efficacy and safety) and above all with minimal interference with their cancer treatment.

A 2018 online survey from 519 patients recruited on the Facebook page of a thrombosis association (10) assessed their preference (about reversibility, biomonitoring, and the age of the treatment) and concern about recurrent VTE and the bleeding. Respondents were predominantly young (mean 45 years) women (83%); 29.9% strongly disagreed with the following statement: “I am comfortable using a blood thinner where the levels cannot be followed.” In terms of reversibility, 52.9% strongly agreed with the statement: “I prefer a blood thinner that is reversible.” Moreover, about the age of treatment, 14.5% strongly agreed with the statement: “I am comfortable using the newest treatment vs. an older but more established treatment” while 23.2% strongly disagreed. Finally, 33.1% reported being extremely concerned about recurrent VTE (and about as moderately concerned), 21.4% were extremely concerned about major bleeding. These results are of limited interest for practice in the general population but we find quite similar results in terms of concerns about disease (63% regardless of the level of concerns). We did not assess in our study the patient's preference regarding reversibility of an anticoagulant, ability to monitor anticoagulant levels or the date of marketing authorization of a treatment. These topics involve pharmacodynamic and pharmacokinetic knowledge that patients do not have, and are still under discussion within the medical community (11). It would be interesting to conduct a qualitative study to formalize hypotheses about patient preferences. Otherwise, our clinical experience leads us to believe that these themes are not relevant to analyze and we have made the choice in our work not to consider these aspects and to say to patients; “These are all good (i.e., recommended) treatments.”

A multicenter study in 2019, granted by Bayer® (12) among 163 patients with cancer and VTE who had already started direct oral anticoagulant treatment by Rivaroxaban assessed preference about (a) route of administration (injection/tablet), (b) frequency of intake (once/twice daily), and (c) need of regular controls of the International Normalized Ratio (INR) at least every 3–4 weeks. The route of administration was by far the most important attribute for a patient's choice (73.8% of the overall decision), over the intake frequency (6.5%) and the biological monitoring (0.6%).

A 2020 meta-analysis has proposed an approach combining quantitative and qualitative analysis (13). Findings from quantitative research studies show high variability due to the diversity of the patient populations included in the studies. Qualitative findings corroborate previous results regarding patients' preferences for oral treatment over injectable treatment. However, all of the included qualitative studies focused on VKAs rather than on DOACs. Finally, quantitative and qualitative findings reveal patients' positive perceptions of treatment and diagnosis when informed and when included in the treatment decision-making process.

The percentage of VTE history in our study (28%) is consistent with the literature (1) but this history does not seem to have an impact on the choice of anticoagulant treatment, whatever the type of treatment previously prescribed. However, our population presents few PE (33%) and many DVT (80%) with also a predominantly male population (59%) and a high hospitalization rate (53%), which limits the extrapolation of these results. The distribution of risk factors corresponds to that expected in a population that includes half of hospitalized patients. When given a choice, patients chose oral treatment without dose adjustment or biological monitoring (74%) and preferred daily intake justified by the ease of use. It should be noted that no patient mentioned the lack of antidote as an obstacle to oral treatment. Factors statistically associated with the preference of injectable treatment was age, polymedication (57% patients who taking more than 3 oral treatment per day chose injectable treatment or had no preference) and a PE-type event. The duration of treatment does not change this choice. We believe that the association between PE and the choice of oral treatment might be secondary to a bias: indeed, the diagnosis of PE probably took place before the appointment with the vascular physician and an oral anticoagulant treatment may have been prescribed or recommended to the patient before inclusion in the study. Indeed, except for cancer, oral treatment is currently recommended as first-line treatment in France for VTE (14). No recanalization or other invasive procedure was proposed in this study. In France, this procedure is not part of routine care in the initial phase of VTE.

### Limitations

The first limitation of this study is its design: a questionnaire read by different physicians, thus data depends on the ability of the interviewer and his or her own biases that could impact the way he or she captures responses. However, we tried to limit the bias related to the physician's influence on the patient by asking the physician to read sample questions without adding precision. Secondly, the patient's choice is evaluated only at the time of diagnosis and no adhesion measurement is performed afterwards. Prospective clinical trials are needed to clarify the evolution of preferences during treatment. Lastly, the response rate was only 16%. Despite the support of the SFMV, it is difficult to assess the representativeness of the respondent population.

## Conclusion

Patients' preference in terms of anticoagulant treatment in thromboembolic venous disease is in favor of oral treatment without adjustment or biomonitoring and with once daily intake. When the prescription of an injectable treatment is chosen, a prolonged duration of treatment does not seem to be a constraint for the patient.

## Data Availability Statement

The raw data supporting the conclusions of this article will be made available by the authors, without undue reservation.

## Ethics Statement

The questionnaire and methodology for this study was approved by the Human Research Ethics Committee of the University of Rennes (Ethics approval number: CPP #8/589). The patients/participants provided their written informed consent to participate in this study.

## Author Contributions

Material preparation, data collection, and analysis were performed by DL and GM. The first draft of the manuscript was written by DL and all authors commented on previous versions of the manuscript. All authors contributed to the article and approved the submitted version.

## Conflict of Interest

GM has received fees as speaker from Bristol-Myers-Squibb, Leo Pharma, GSK, and Bayer. DL has received fees as speaker from Bristol-Myers-Squibb, Leo Pharma, and Bayer. The remaining authors declare that the research was conducted in the absence of any commercial or financial relationships that could be construed as a potential conflict of interest.

## The SFMV VTE Study Group

Karpoff Sandrine, Accassat Sandrine, De Bortoli Marc, Girard Françoise, Jurus Christine, Chleir Franck, Pottier Camille, Bouchereau Robert, Demarest Michèle, Bauvin Nathalie, Petitalot Maxime, Radureau Chloé, Bonnin-Vaeze Chantal, Milimo Raoul, Diard Antoine, Guillet Mathieu, Khider Lina, Mirault Tristan, Galloula Alexandre, Mortelette Hélene, Blin Emmanuel, Laffont Joëlle, Sindres Pierre, Purser Christine, Bensedrine Sophia, Trihan Jean-Eudes, Monthé Béranger, Charasson Marie, Philippe Landreau.

## Supplementary Material

Appendix 1 Questionnaire.

Appendix 2 Risk factors for Venous Thromboembolic disease.
