# Supplementary material for: Patient Characteristics and Preferences Regarding Anticoagulant Treatment in Venous Thromboembolic Disease
Source: Front Cardiovasc Med. 2021 Jun 21;8:675969. doi: 10.3389/fcvm.2021.675969 (PMC8255622; doi:10.3389/fcvm.2021.675969)
Supplement: Appendix 1 — Questionnaire. [file Data_Sheet_1.docx]

**Please read this text to your patient if he or she has venous thromboembolic disease (any type): *"You have venous thrombosis or "phlebitis" and/or pulmonary embolism, i.e. the presence of a blood clot blocked in a vessel. This requires a treatment that makes the blood thinner. Within the framework of a study made in France, if you agree, I am going to ask you simple questions for less than 5 minutes, which will have no consequence on the treatment that will be prescribed to you; you can answer what you were thinking in concern, freely and spontaneously.”***

**Do not provide additional information to avoid bias in the results.**

1. Have you ever had?

- pulmonary embolism
- deep vein thrombosis (or “phlebitis”)
- pulmonary embolism and Deep vein thrombosis (or “phlebitis”)
- superficial vein thrombosis

1. How were you treated during these previous episodes?

- per tablets or pills (specify the approximate number of days): ………………
- per injection (specify the approximate number of days): …………………….
- both (specify the approximate number of days): ……………………………..

1. Do you know people who have had venous thrombosis, phlebitis or pulmonary embolism?

- yes
- no

1. Are you concerned about having a venous thrombosis, phlebitis or pulmonary embolism?

- yes
- no

1. How much medication are you taking? ……………………….
2. Do you feel over-medicated?

- yes
- no

**Please read this text: *“There are two types of treatment: tablets or subcutaneous injections with needle made by yourself or a nurse. These are all good treatments”***

1. What type of treatment would you prefer if you could choose?

- fixed-dose tablet that does not require a blood test
- tablet with dose adjustment and blood monitoring
- subcutaneous injection
- it doesn't matter

1. In case of oral treatment, which number of doses per day do you prefer?

- twice a day
- once a day
- no preference

1. Why?

- more efficient
- more practical
- I am afraid of injections
- I have too many injections
- forget to take
- I do not like injections
- other: …………….

1. If injectable treatment, number per day:

- twice a day
- once a day
- no preference

1. Why?

- more efficient
- more practical
- I am afraid of pills
- I have too many pills
- forget to take
- I do not like pills
- other: …………….

1. If the treatment lasts

|  | I prefer tablets | I prefer injections | It Doesn't Matter |
| --- | --- | --- | --- |
| 6 weeks | O | O | O |
| 3 months | O | O | O |
| 6 months | O | O | O |
| more than 6 months | O | O | O |
