# Supplementary material for: Patient Characteristics and Preferences Regarding Anticoagulant Treatment in Venous Thromboembolic Disease
Source: Front Cardiovasc Med. 2021 Jun 21;8:675969. doi: 10.3389/fcvm.2021.675969 (PMC8255622; doi:10.3389/fcvm.2021.675969)
Supplement: Appendix 2 — Risk factors for Venous Thromboembolic disease. [file Data_Sheet_2.docx]

**Annexe 2.** Risk factors for Venous Thromboembolic disease

1. VTE provoked by a persisting risk factor:

- active cancer (i.e. cancer at baseline (diagnosis or treatment < 6 months or recurrent or metastatic cancer)), excluding basal-cell or squamous cell carcinoma

- inflammatory bowel disorders

- thrombophilia including deficiency of antithrombin, protein C, or protein S, factor V Leiden or prothrombin gene mutation, and antiphospholipid syndrome

- varicose veins

2. VTE provoked by a transient risk factor if within 3 months :

- major surgery or major trauma

- immobilization due to a medical disorder

- prolonged travel > 8 hours

- use of hormonal therapy

- pregnancy or puerperium

- leg injury with impaired mobility, including orthopedic surgery/ arthroscopy

- catheter related thrombosis

3. Unprovoked VTE: these are all other situations.
